# Molecular targets in bone cancer pain: a systematic review of inflammatory cytokines

**DOI:** 10.1007/s00109-024-02464-2

**Published:** 2024-06-28

**Authors:** Jacinta Ruivo, Isaura Tavares, Daniel H. Pozza

**Affiliations:** 1 Experimental Biology Unit, Department of Biomedicine, Faculty of Medicine of Porto, University of Porto, 4200-319 Porto, Portugal; 2 Institute for Research and Innovation in Health and IBMC, University of Porto, 4200-135 Porto, Portugal

**Keywords:** Bone neoplasms, Pain, Intractable, Cytokines, Inflammation, Molecular targeted therapy, Quality of life

## Abstract

Bone cancer pain (BCP) profoundly impacts patient’s quality of life, demanding more effective pain management strategies. The aim of this systematic review was to investigate the role of inflammatory cytokines as potential molecular targets in BCP. A systematic search for animal rodent models of bone cancer pain studies was conducted in PubMed, Scopus, and Web of Science. Methodological quality and risk of bias were assessed using the SYRCLE RoB tool. Twenty-five articles met the inclusion criteria, comprising animal studies investigating molecular targets related to inflammatory cytokines in BCP. A low to moderate risk of bias was reported. Key findings in 23 manuscripts revealed upregulated classic pro-inflammatory cytokines (TNF-α, IL-1β, IL-6, IL-17, IL-18, IL-33) and chemokines in the spinal cord, periaqueductal gray, and dorsal root ganglia. Interventions targeting these cytokines consistently mitigated pain behaviors. Additionally, it was demonstrated that glial cells, due to their involvement in the release of inflammatory cytokines, emerged as significant contributors to BCP. This systematic review underscores the significance of inflammatory cytokines as potential molecular targets for alleviating BCP. It emphasizes the promise of targeted interventions and advocates for further research to translate these findings into effective therapeutic strategies. Ultimately, this approach holds the potential to enhance the patient’s quality of life.

## Introduction

Bone cancer pain (BCP) is a prevalent and distressing type of pain associated with advanced malignancies, mainly from metastasis to the bone, but also from primary bone tumors. BCP can be categorized into ongoing pain, spontaneous breakthrough pain, and movement-evoked breakthrough pain. Ongoing pain, often an initial symptom, starts as a dull and constant throbbing sensation that intensifies over time. As bone cancer progresses, individuals may experience intermittent episodes of intense pain, occurring spontaneously, referred to as spontaneous breakthrough pain, or, more frequently, in response to weight-bearing or movement of the affected bone, known as movement-evoked breakthrough pain [1, 2].

While cancer patients are experiencing longer lifespans due to the advances in diagnostics and treatments, the side effects, which include pain, are substantially compromising the overall quality of life for these individuals [3]. More than half of patients with bone metastasis or advanced osteocarcinoma are undertreated for their BCP, experiencing daily moderate to severe pain [4, 5]. Currently, BCP is largely managed based on the World Health Organization’s “analgesic ladder” [6]. In addition, other adjuvant therapies including antiepileptics, steroids, antidepressants, radiotherapy, nerve blocks, nerve lesions, and surgery are often used to control cancer pain [4, 7, 8]. This approach faces several limitations due to tolerance, side effects, and inadequate analgesia [9]. Additionally, BCP is inadequately managed partly due to the current insufficient understanding of the specific mechanisms underlying this pain [10, 11].

**Fig. 1:**
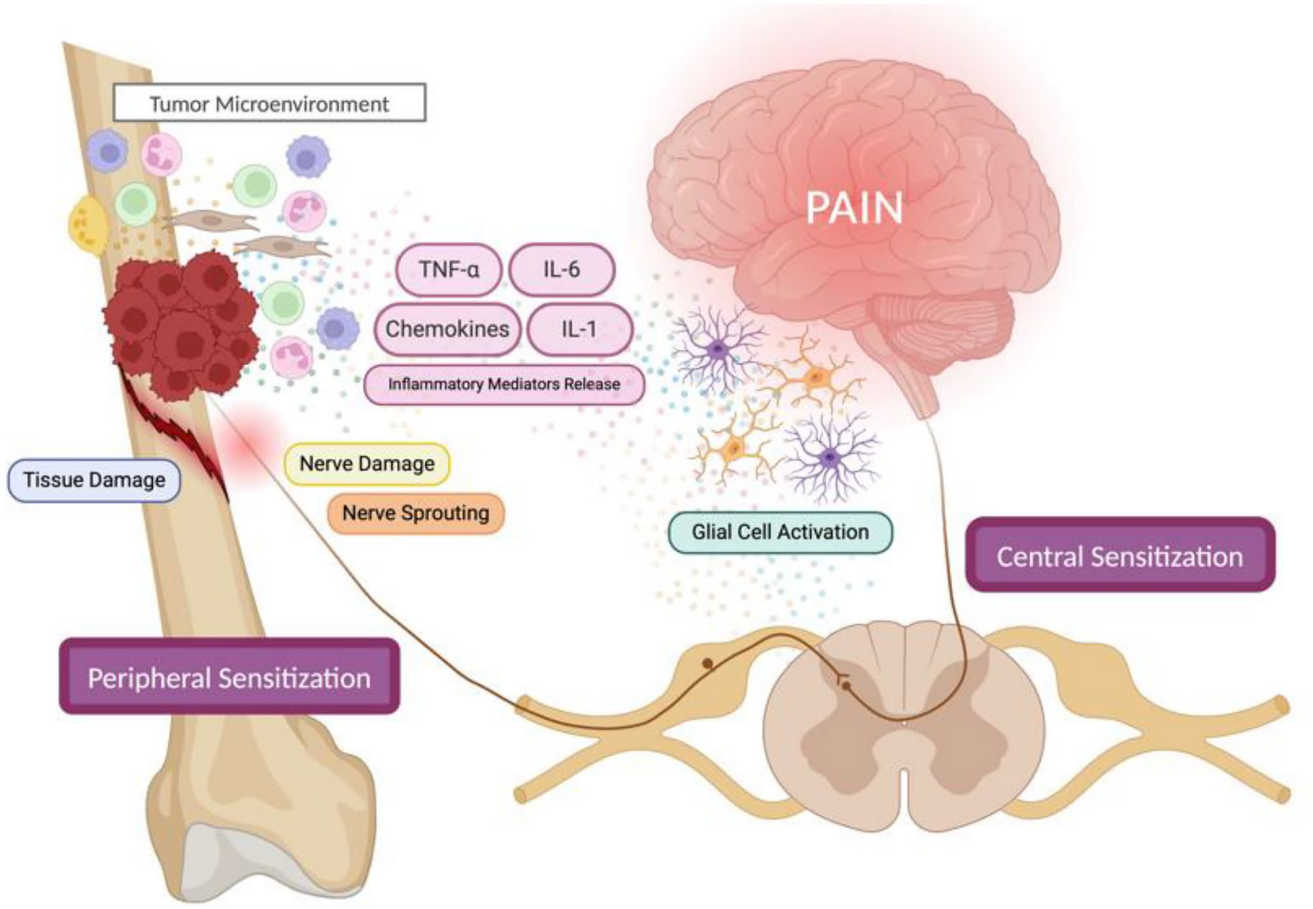
Schematic representation of the mechanisms behind BCP. The release of pronociceptive factors by tumor and immune cells, direct tissue damage, and bone degradation through osteoclast (yellow) activation leads to the activation of nociceptors and the perception of pain. If prolonged over time, continuous nociceptive stimuli can lead to peripheral sensitization, with neuronal damage and ectopic appearance of nerve fibers. Because of peripheral events, central excitability changes happen, such as microglial activation and inflammatory mediator release, leading to central sensitization

Chronic pain is influenced by physical, social, emotional, cognitive, environmental, and behavioral factors. Its transition from acute to chronic involves complex mechanisms like peripheral and central sensitization, increasing pain perception. In bone cancer pain (BCP), tumor-induced inflammation, nerve reprogramming, and peripheral nerve sensitization play key roles. The daunting nature of BCP arises from the complex interplay of nociceptive, neuropathic, and associated inflammatory mechanisms that evolve and change with disease progression (Fig. 1). The tumor mass comprises several types of cells, such as tumor cells, macrophages, neutrophils, T-lymphocytes, fibroblasts, and endothelial cells that secrete a variety of mediators, including inflammatory cytokines such as TNF-α, IL-1, and IL-6 [12]. Bone nociceptors are sensitized by these mediators, thereby initiating and perpetuating pain. The continued stimulation of the sensory nerve fibers can drive ectopic sprouting of nerve fibers and neuroma formation, leading to peripheral sensitization. Glial cell activation and subsequent release of inflammatory mediators in the central nervous system contribute to neuronal hypersensitive states [10, 11, 13]. If not properly managed, it can evolve to central sensitization and the installation of chronic pain, which is very difficult to manage [14]. Targeting specific cytokines or their receptors presents a promising, tailored approach to mitigating BCP, overcoming some limitations typically associated with traditional management.

Currently, most of the research on BCP relies on rodent models of BCP. These models are typically created through inoculation of tumor cells, such as Walker 256 mammary gland carcinoma cells, fibrosarcoma NCTC 2472 cells, or prostate cancer cells, into the bone marrow, mainly of tibia and femur. BCP has been modeled in many domestic animals; however, rodent models predominate in BCP preclinical studies. These models allow investigators to correlate cancer-induced bone remodeling, pain behavioral signs, and neurochemical changes in the spinal cord and primary afferent neurons. Pain is a subjective experience, so animal models often rely on observing pain-related behaviors, like reflex responses to a given stimulus, such as mechanical (e.g., paw withdrawal threshold to von Frey filaments) or thermal (e.g., hot plate test) stimuli. Additionally, spontaneous activities like limb use, flinching, or guarding time can be evaluated. These pain-related behaviors seem to mirror the complex clinical manifestations of ongoing pain, movement-evoked pain, and spontaneous breakthrough pain [15–18].

The present systematic review aimed to provide a comprehensive assessment of the current state of knowledge regarding potential molecular targets in rodent models within the domain of inflammatory cytokines in the context of BCP. This effort potentially enables the development of targeted cytokine-based therapeutic strategies for the management of BCP, ultimately improving the quality of life for individuals facing with this challenging symptomatology.

## Materials and methods

This systematic review adhered to the Preferred Reporting Items for Systematic Reviews and Meta-Analyses (PRISMA) 2020 guidelines [19]. The PICO question was: In animal models of bone cancer pain (P), how does the implementation of targeted cytokine-based therapeutic strategies (I) compared to control groups without cytokine modulation (C) influence pain behavior (O)?

For this review, only publications including rodent animal models of BCP were evaluated, focusing primarily on studies investigating the role of inflammatory cytokines as potential molecular targets. The included outcomes of interest were pain behavior and the identification of potential molecular targets.

Studies not published in English, those involving animal models other than rodents, review articles, case reports, and studies not providing relevant data on inflammatory cytokines were excluded. Duplicate publications were also omitted from consideration.

To gather relevant studies, we systematically searched three electronic databases, namely PubMed, Scopus, and Web of Science, starting on November 23, 2022, and retrieving all articles published until October 10, 2023. For the PubMed research, the following Mesh terms were used: “Cytokines” [Mesh] AND “Cancer Pain” [Mesh]. For Web of Science, the keywords were “cytokines” AND “cancer pain”. Finally, the Scopus search for articles used was TITLE-ABS-KEY (“cytokine” AND “cancer pain” AND (“target” OR “treatment”) AND (“animal model” OR “animal study” OR “animal experiment” OR “animal” OR “rat” OR “mice”)).

Screening of titles and abstracts of identified records was carried out independently by two reviewers to determine eligibility. Full texts of potentially relevant studies were then assessed for a comprehensive analysis. The level of agreement between the authors was assessed using the Kappa test.

Data from eligible studies was extracted. The information collected from each study included information about the study’s authors, publication year, species and characteristics of the animals used, BCP model, molecular mechanisms investigated, experimental interventions, pain behavior assessments, and other outcome measures such as molecular expression and key findings. Data collection involved two independent reviewers, reaching a consensus on discrepancies.

A narrative synthesis was conducted to summarize the findings of the included studies. Given the expected heterogeneity in animal models and outcome measures, it was decided to present a qualitative synthesis instead of a meta-analysis. To assess the methodological quality and risk of bias in the included studies, the SYRCLE RoB tool was employed, specifically designed for animal studies.

## Results

**Fig. 2:**
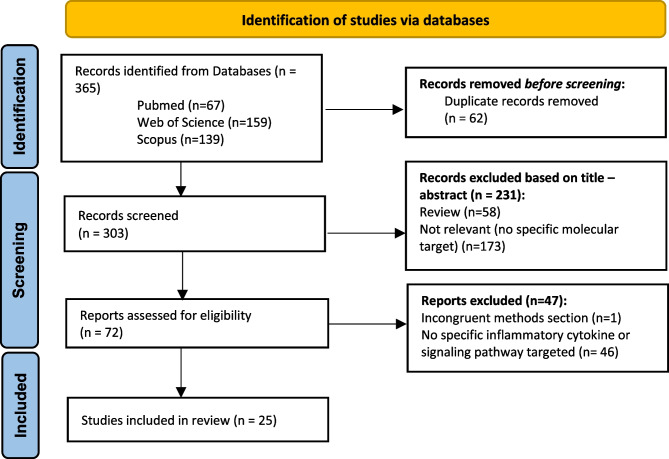
Flow diagram outlining included studies selection

A comprehensive literature search in PubMed, Web of Science, and Scopus databases resulted in 365 potential records identified. Following the removal of duplicate records, 303 records remained for the title and abstract review. After the analysis of the title and abstract, 72 articles were selected for full-text examination. The final inclusion criteria were met by 25 articles, comprising animal experiments investigating potential molecular targets related to inflammatory cytokines in BCP. Figure 2 shows the entire literature search process. The Kappa coefficient for interrater agreement was 1, indicating perfect agreement between the reviewers.

### Characteristics of included studies

Of the selected 25 articles, two kinds of rodents were included: rats (*n* = 22) [20–41] and mice (*n* = 3) [42–44]. Regarding their strains, Sprague–Dawley (SD) rats (*n* = 17) [20, 23–38], Wistar rats (*n* = 3) [21, 22, 39], Copenhagen rats (*n* = 1) [40], Fisher F344/NHsd rats (*n* = 1) [41], C3H/HeN mice (*n* = 1) [42], BALB/c mice (*n* = 1) [43], C57Bl/6J mice (*n* = 1) [44], and transgenic mice (*n* = 2) [43, 44] were included. Twenty studies selected female animals [20, 23–39] [41] [43], while five studies selected male animals [21, 22, 40, 42, 44]. Regarding the BCP model, the majority of studies (*n* = 20) conducted an intramedullary injection of Walker 256 rat breast cancer cells into the tibia [20–39]. The summary of key findings is depicted in Table 1.

**Table 1:** Comprehensive overview of the key characteristics of the included studies

**Reference**	**Species (age, sex, weight)**	**Cell line, site**	**Molecular targets investigated**	**Experimental interventions (molecules tested)**	**Pain behavior assessments**	**Molecular expression** **Other outcome measures**	**Key findings**
[20]	SD rats (female, 200–220 g)	Walker 256 TIBIA	TNF-α	*Intrathecal administration* • solTNF inhibitor, XPro1595	▪ PWT	*Spinal cord L4-L5* TNF-α, IL-1β, IL-6, p-ERK, p-p38, p-JNK, ERK, p38, JNK (WB) Iba-1, GFAP, NeuN, p-p38 (IF)	*After TCI* ↑ TNF-α, IL-1β, IL-6 ↑ p-ERK, p-p38, p-JNK ↑ Iba-1, GFAP (microglia and astrocyte activation) ↑ mechanical allodynia *After blocking solTNF* ↓ TNF-α, IL-1β, IL-6 ↓ p-p38 ↓ microglia and astrocyte activation ↓ mechanical allodynia
[21]	Wistar rats (adult, male, 200–250 g)	Walker 256 TIBIA	IL-1β IL-6 TNF-α PI3K/Akt/mTOR	*PAG infusion* • mTOR inhibitor rapamycin • PI3K inhibitor LY294002 • IL-1β receptor antagonist IL-1Ra • SC144, an inhibitor to complexed IL-6R-gp130 • TNF-α antagonist etanercept	▪ PWT ▪ PWL	*dlPAG* IL-1β, IL-6 and TNF-α (ELISA) IL-1R, IL-6R and TNFR1, p-PI3K, p-Akt, p-mTOR, p-S6K1, PI3K, Akt, mTOR (WB)	*After TCI* ↑ TNF-α, IL-1β, IL-6 and IL-1R, IL-6R, TNFR1 ↑ p-PI3K, p-Akt, p-mTOR ↑ mechanical allodynia and thermal hyperalgesia *After blocking PI3K* ↓ mechanical and thermal hyperalgesia *After blocking mTOR* ↓ mechanical and thermal hyperalgesia *After blocking PIC* ↓p-PI3K, p-mTOR and p-S6K1 ↓ mechanical allodynia and thermal hyperalgesia
[22]	Wistar rats (adult, male, 200–250 g)	Walker 256 TIBIA	IL-6 TNF-α TRPA1	*Intraperitoneal administration* • TNF-a synthesis inhibitor, pentoxifylline (PTX) • SC144, an inhibitor to complexed IL-6R-gp130 • TRPA1 antagonist HC030031	▪ PWT ▪ PWL	*DRG L4–L6* TNF-α and IL-6 (ELISA) TRPA1, TNFR1, IL-6R, p-p38-MAPK, p38-MAPK, p-JNK, JNK (WB)	*After TCI* ↑ TNF-α, IL-6, TNFR1, TRPA1 ↑ p-p38-MAPK, p-JNK ↑ mechanical allodynia and thermal hyperalgesia *After blocking TRPA1* ↓ mechanical allodynia and thermal hyperalgesia *After blocking TNF-α, IL-6 (individually)* ↓ TRPA1, p38-MAPK, and p-JNK ↓ mechanical allodynia and thermal hyperalgesia
[23]	SD rats (adult, female, 160–180 g)	Walker 256 TIBIA	IL-18	*Intrathecal administration* • IL-18 binding protein • anti–IL-18R • recombinant rat IL-18 • NMDA receptor blocker MK801 • Src kinase-specific inhibitor PP1	▪ PWT ▪ PWL ▪ SG ▪ SF ▪ NPG ▪ LUS	*Spinal cord L4-L5* IL-18, IL-18R, pNR2B, p-CREB, pPKCγ, GluN2B, p-GluN2B (WB) IL-18, IL-18R, GluN2B, NeuN, GFAP, Iba1 (IF)	*After TCI* ↑ IL-18, IL-18R ↑ p-CREB, pPKCγ, p-GluN2B ↑ Iba-1, GFAP (microglia and astrocyte activation) ↑ bone cancer-related pain behaviors *After blocking IL-18* ↓ p-CREB, pPKCγ, p-GluN2B ↓ microglia and astrocyte activation ↓ bone cancer-related pain behaviors *After IL-18 administration* ↑ p-GluN2B (GluN2B and IL-18 colocalized) ↑ bone cancer-related pain behaviors
[24]	SD rats (adult, female, 180–200 g)	Walker 256 TIBIA	IL-24	*Injected subcutaneously* • adenovirus-mediated IL-24 gene transfer vector (Ad-IL-24)	▪ PWT	*Tibia bones* β-EP and IL-6 (ELISA) Bone histology	*After TCI* ↑ IL-6 ↓ β-EP ↑ mechanical allodynia *After Ad-IL-24 administration* ↓ tumor growth ↓ IL-6 ↑ β-EP ↓ mechanical allodynia
[25]	SD rats (female, 180–200 g)	Walker 256 TIBIA	GM-CSF	*Injected in the vicinity of the tibia bone* • antibody against GM-CSF • antibody GM-CSFR • GM-CSF competitive antagonist E21R	▪ PWT ▪ PWL		*After TCI* ↑ mechanical allodynia and thermal hyperalgesia *After blocking GM-CSF* *(anti-GM-CSF, anti-GM-CSFR and antagonist)* ↓ mechanical allodynia and thermal hyperalgesia (anti-GM-CSFR with stronger effect)
[26]	SD rats (adult, female, 180–200 g)	Walker 256 TIBIA	MCP-1/CCR2 PI3K	*Intrathecal administration* • exogenous recombinant MCP-1 • CCR2 antagonist RS-504393 • PI3K inhibitor LY 294002	▪ PWT	*Spinal cord L4-L5* P2X4R, p-Akt, MCP-1 (WB)	*After TCI* ↑ MCP-1, P2X4R, p-AKT ↑ bone cancer-related pain behaviors *After MCP-1 administration (naïve rats)* ↑ mechanical allodynia *After blocking CCR2* ↓ P2X4R, p-Akt ↓ mechanical allodynia *After blocking PI3K* ↓P2X4R ↓ mechanical allodynia
[27]	SD rats (adult, female, 180–200 g)	Walker 256 TIBIA	MCP-1/CCR2 NF-kB	*Intrathecal administration* • MCP-1 neutralizing antibody • CCR2 selective antagonist RS504393 • NF-kB inhibitor BAY11–7082	▪ PWT ▪ SFI	*Spinal cord L3-L5* MCP-1, CCR2, NF-kB, p-NF-kB, IL-4, TNF-α, IFN-γ (WB) MCP-1, CCR2, NF-kB, GFAP, CD11b, NeuN (IF) MCP-1, CCR2 (RT-PCR)	*After TCI* ↑ MCP-1, CCR2 (neurons ++) ↑ NF-kB, p-NF-kB (neurons ++) ↑ IL-4, TNF-α, IFN-γ ↑ bone cancer-related pain behaviors *After blocking MCP-1 or CCR2 (individually)* ↓ TNF-α, IFN-γ ↑ IL-4 ↓ bone cancer-related pain behaviors *After blocking NF-kB* ↓MCP-1 and CCR2 ↓ TNF-α, IFN-γ ↑ IL-4 ↓ bone cancer-related pain behaviors
[28]	SD rats (adult, female, 180–200 g)	Walker 256 TIBIA	CXCL1/CXCR2 NFκB	*vlPAG cannulation and microinjections* • CXCL1 neutralizing antibody • exogenous CXCL1 • CXCR2 antagonist SB225002 • NF-kB inhibitor BAY11–7082 • astrocytic cytotoxin, l-α-aminoadipic acid	▪ PWT	*vlPAG* p-NF-kB, CXCL1, and CXCR2 (RT-PCR, WB, IF) GFAP, NeuN, CD11 (IF)	*After TCI* ↑ CXCL1 (astrocytes ++), CXCR2 (neurons ++) ↑ pNF-kB (astrocytes, neurons ++) ↑ GFAP (astrocyte activation) ↑ mechanical allodynia *After CXCL1 administration* ↑ mechanical allodynia *After blocking CXCL1* ↓ mechanical allodynia *After blocking CXCR2* ↓ mechanical allodynia ↓ mechanical allodynia in CXCL1-treated rats *After blocking NF-kB or astrocytes (individually)* ↓ CXCL1 ↓ mechanical allodynia
[29]	SD rats (adult, female, 160–200 g)	Walker 256 TIBIA	CXCL1 JNK/CXCL1	*Intrathecal administration* • JNK inhibitor SP600125 • CXCL1 neutralizing antibody	▪ PWT ▪ PWL	*Spinal cord L4-L5* p-JNK and CXCL1 (WB, IHC) CXCL1 (RT-PCR)	*After TCI* ↑ CXCL1, p-JNK ↑ mechanical allodynia and thermal hyperalgesia *After blocking CXCL1* ↓ mechanical allodynia and thermal hyperalgesia *After blocking JNK* ↓ CXCL1 ↓ mechanical allodynia and thermal hyperalgesia
[30]	SD rats (female, 180–200 g)	Walker 256 TIBIA	CXCL10/CXCR3	*Intrathecal administration* • recombinant rat CXCL10 protein (rrCXCL10) • anti-CXCL10 neutralizing antibody • AMG487, an antagonist of CXCR3 • minocycline hydrochloride (inhibitor of microglia)	▪ PWT	*Spinal cord L3-L5* CXCL10 and CXCR3 (RT-PCR) CXCL10, CXCR3, CD11b (IF)	*After TCI* ↑ CXCL10, CXCR3 ↑ CD11b (microglia activation) ↑ mechanical allodynia *After CXCL10 administration* ↑ mechanical allodynia *After blocking CXCL10* ↓ mechanical allodynia *After blocking CXCR3* ↓ microglia activation ↓ mechanical allodynia *After inhibiting microglia* ↓ CXCL10 ↓ mechanical allodynia in rrCXCL10-treated rats
[31]	SD rats (adult, female, 180–220 g)	Walker 256 TIBIA	CXCL12/CXCR4 RhoA/ROCK2	*Intrathecal administration* • ROCK2 inhibitor (Fasudil, downstream of RhoA) • CXCR4 inhibitor plerixafor (AMD3100) • Rat recombinant SDF-1 (CXCL12)	▪ 50% PWT ▪ PWL	*Spinal cord L4-L6* CXCR4, p-RhoA, p-ROCK2 (WB, IF) NeuN, GFAP, Iba-1 (IF)	*After TCI* ↑ CXCR4, p-RhoA, p-ROCK2 (colocalized, neurons ++) ↑ bone cancer-related pain behaviors *After CXCL12 administration* ↑ p-RhoA, p-ROCK2 ↑ mechanical allodynia and thermal hyperalgesia *After blocking CXCR4* ↓ CXCR4, p-RhoA, p-ROCK2 ↓ mechanical allodynia and thermal hyperalgesia *After blocking ROCK2* ↓ p-ROCK2, p-RhoA ↓ mechanical allodynia and thermal hyperalgesia *After blocking CXCR4 or ROCK2 in CXCL12-treated rats* ↓ p-RhoA, p-ROCK2
[32]	SD rats (adult, female, 180–200 g)	Walker 256 TIBIA	CXCL12/CXCR4 JNK	*Intrathecal administration* • JNK inhibitor SP600125 • Anti-CXCL12 neutralizing antibody • Astrocytic inhibitor fluorocitrate • CXCR4 inhibitor AMD3100	▪ PWT ▪ PWL	*Spinal cord, DRG L4-5* CXCL12 and CXCR4 (WB, IF) c-FOS, NeuN, GFAP, Iba-1 (IF)	*After TCI* ↑ CXCL12 (astrocytes ++), CXCR4 ↑ c-Fos, NeuN, GFAP, Iba-1 (neuron and glia activation) ↑ mechanical allodynia and thermal hyperalgesia *After blocking JNK or inhibiting astrocytes* ↓ CXCL12 ↓ mechanical allodynia and thermal hyperalgesia *After blocking CXCL12* ↓ mechanical allodynia and thermal hyperalgesia *After blocking CXCR4* ↓ neuron and glia activation ↓ mechanical allodynia and thermal hyperalgesia
[33]	SD rats (adult, female, 180–220 g)	Walker 256 TIBIA	CXCL12/CXCR4 CaMKII PLC	*Intrathecal administration* • CaMKII inhibitor AIP2 • CXCR4 inhibitor Plerixafor (AMD3100) • PLC inhibitor U73122 • Rat recombinant SDF-1 (CXCL12) • Small interfering RNA against CXCR4	▪ 50% PWT ▪ PWL ▪ SF ▪ LUS	*Spinal cord L4-L6* CXCR4, p-CaMKII, CaMKII, p-CREB, CREB, NMDAR1 (WB, IF, RT-PCR) NeuN, GFAP, IBA-1 (IF)	*After TCI* ↑ CXCR4, p-CaMKII, p-CREB, NMDAR1 (colocalized, neurons++) ↑ bone cancer-related pain behaviors *After CXCL12 administration* ↑ p-CaMKII, p-CREB ↑ mechanical allodynia and thermal hyperalgesia *After blocking CXCR4 or PLC in CXCL12-treated rats* ↓ p-CaMKII, p-CREB ↓ mechanical allodynia and thermal hyperalgesia *After blocking CaMKII in CXCL12-treated rats* ↓ mechanical allodynia and thermal hyperalgesia *After blocking CXCR4* ↓ NMDAR1 (neurons + +) ↓ mechanical allodynia
[34]	SD rats (adult, female, 180–220 g)	Walker 256 TIBIA	CXCL13/CXCR5 p38 ERK AKT	*Intrathecal administration* • murine recombinant CXCL13 • small interfering RNA targeting CXCR5 • morphine • p38 inhibitor SB203580 • ERK1/2 inhibitor PD98059 • AKT inhibitor	▪ PWT	*Spinal cord L1-L5* Akt, p38, ERK1/2, p-Akt, p-p38, p-ERK1/2 CXCL13 and CXCR5 (WB)	*After TCI* ↑ CXCL13, CXCR5 ↑ p-p38, p-ERK and p-Akt ↑ mechanical allodynia *After CXCL13 administration* ↑ p-p38, p-ERK and p-Akt ↑ mechanical allodynia ↓ morphine analgesia *After morphine administration* ↓ TNF-α, IL-1β, IL-6 ↓ p-p38, p-ERK and p-Akt ↓ mechanical allodynia *After blocking CXCR5* ↓ CXCR5 ↓ p-p38, p-ERK and p-Akt ↓ mechanical allodynia *After blocking p38, ERK and AKT (individually)* ↓ p-p38, p-ERK, and p-Akt (respectively) ↓ mechanical allodynia ↑ morphine analgesia
[35]	SD rats (adult, female, 180–200 g)	Walker 256 TIBIA + rat BCP-morphine tolerance model (9 days of intrathecally administering morphine) TIBIA	CXCL13	*Intrathecal administration* • rmCXCL13 • neutralizing anti-CXCL13	▪ PWT ▪ MWD	*Spinal cord L4-L6* CXCL13 (RT-PCR, WB, IF) NeuN (IF)	*After TCI* + *induced morphine tolerance* ↑ CXCL13 (neurons ++) ↑ mechanical allodynia *After CXCL13 administration* ↑ mechanical allodynia ↓ morphine analgesia *After blocking CXCL13* ↓ mechanical allodynia ↑ morphine analgesia
[36]	SD rats (female, 160–180 g)	Walker 256 TIBIA	SOCS3	*Intrathecal administration* • recombinant LV vectors LV-SOCS3	▪ 50% PWT ▪ PWL ▪ WBD	*DRG L2-L5* SOCS3 and TLR4 (WB, IF) β-tubulin, CD11b, and GFAP (IF) *Whole-cell patch clamping*	*After TCI* ↓ SOCS3 ↑ TLR4 ↑ excitability of DRG neurons innervating the tibia ↑ bone cancer-related pain behaviors *After SOCS3 overexpression* ↓ TLR4 ↓ hyperexcitability of DRG neurons innervating the tibia ↓ bone cancer-related pain behaviors
[37]	SD rats (female, 180–200 g)	Walker 256 TIBIA	NLRP3 inflammasome	*Intraperitoneal administration* • NLRP3 inhibitor MCC950	▪ PWT	*Spinal cord L4-L6* NLRP3, ASC, Caspase-1, IL-1β, NLRP1, IL-18, TNF-α (WB) NLRP3, ASC, Caspase-1, IL-1β, GFAP, Iba1, NeuN (IF)	*After TCI* ↑ NLRP3, ASC, Caspase-1, NLR1 ↑ IL-1β, IL-18, and TNF-α ↑ mechanical allodynia *After blocking NLPR3* ↓ NLRP3, ASC, Caspase-1 ↓IL-1β, IL-18, and TNF-α ↓ mechanical allodynia
[38]	SD rats (adult, female, 180–200 g)	Walker 256 TIBIA	HMGB1 PKC	*Intrathecal administration* • PKC inhibitor Gö6983 • PKC activator PMA • anti-HMGB1 neutralizing antibody • recombinant human HMGB1	▪ PWT	*Spinal cord L3-L5* HMGB1 (qPCR) HMGB1, RAGE, TNF-α, IL-1β (ELISA) HMGB1, p-HMGB1, p-PKC, RAGE, TNF-α, IL-1bβ (WB) HMGB1, p-PKCα, Iba1, NeuN, GFAP (IF)	*After TCI* ↑ HMGB, p-PKC, RAGE, IL-1β and TNF-α ↑ mechanical allodynia Cytoplasmic HMGB1 and nuclear p-PKC are coexpressed HMGB1 translocation and PKC activation primarily occurred in neurons *After administration of HMGB1* ↑ RAGE, IL-1β, and TNF-α ↑ mechanical allodynia *After blocking HMGB1* ↓ RAGE, IL-1β, and TNF-α ↓ mechanical allodynia *After activating PKC* ↑ PKC, HMBG1, IL-1β, and TNF-α ↑ mechanical allodynia *After blocking PKC* ↓PKC, HMBG1, IL-1β, and TNF-α ↓ mechanical allodynia
[39]	Wistar rats (adult, female, 180–200 g)	Walker 256 TIBIA	HMGB1	*Intrathecal administration* • polyclonal neutralizing antibodies against HMGB1	▪ 50% PWT	*Spinal cord lumbar SDH* HMGB1, IL-1β (WB)	*After TCI* ↑ HMGB1 ↑ IL-1β ↑ mechanical allodynia *After blocking HMGB1* ↓IL-1β ↓ mechanical allodynia
[40]	Copenhagen rats (male, 200–229 g)	AT-3.1 TIBIA	IL-1β	*Intrathecal administration* • IL-1RI receptor antagonist anakinra	▪ PWT	*Spinal cord L4-L6* IL-1β and p-NR1 (WB) IL-1RI and NR1 (IF)	*After TCI* ↑ IL-1β, p-NR1 ↑ mechanical allodynia *After blocking IL-1R* ↓ p-NR1 ↓ mechanical allodynia IL-1RI was localized in NMDAR-containing neurons
[41]	Fisher F344/NHsd rats (female, 150-200 g)	Rat 13,762 MAT B III TIBIA	IL-6	*subcutaneous osmotic mini-pumps* • TB-2–081, IL-6 signaling antagonist of (acute vs sustained administration)	▪ PWT ▪ CCP	*Bone exudates, plasma* IL-6 (ELISA) Bone radiographs and histology (hematoxylin–eosin stain)	*After TCI* ↑ IL-6 ↑ bone remodeling ↑ bone cancer-related pain behaviors *After blocking IL-6 (acute, day 12)* ↓ mechanical allodynia (but not ongoing pain, CCP) *After blocking IL-6 (sustained, start on the day of TCI)* ↓ tumor-induced bone remodeling ↓ bone cancer-related pain behaviors
[42]	C3H/HeN mice (male, 20–25 g)	NCTC 2472 FEMUR	IL-17	*Intrathecal administration* • IL-17/IL-17A antibody	▪ PWT ▪ SF	*Spinal cord L3-L5* IL-17, IL-17A, TGF-β, IL-6, IL-23, IL-17AR (WB) CD3, CD4, IL-17, IL-17A, Foxp3, IL-17AR, GFAP, Iba-1 (IF) IL-17 and IL-17A (ELISA)	*After TCI* ↑ T cell infiltration, Th17/Treg ration ↑ IL-17, IL-17A, IL-17R (microglia ++) ↑ TGF-β, IL-6, and IL-23 ↑ Iba-1 (microglia activation) ↑ bone cancer-related pain behaviors *After blocking IL-17* ↓ Th17/Treg infiltration ↓ microglia activation ↓ bone cancer-related pain behaviors
[43]	BALB/c mice (female, 18–25 g) WT and ST2ko	4T1 FEMUR	IL-33 ST2 (ILR1R)	*Intrathecal administration* • ST2 antibody	▪ PWT ▪ PWL ▪ LUS	*Spinal cord L4-L6* IL-33, ST2 (RT-PCR, WB) IL-1β, IL-6, and TNF-α (Bio-Plex assay) IL-33, NeuN, GFAP, CD11b (IF) Bone radiographs and histology	*After TCI* ↑ IL-33 (astrocytes ++) ↑ IL-1β, IL-6, and TNF-α ↑ bone cancer-related pain behaviors *After blocking ST2* ↓ mechanical allodynia and thermal hyperalgesia *In ST2ko mice* ↓ bone cancer-related pain behaviors
[44]	C57Bl/6 J mice (adult, male, 20 –30 g), WT, TNFR1ko, TNFR2ko, TNFR1 + 2ko	MC57G FEMUR	TNFR1 and TNFR2	*Intraperitoneal administration* • TNF-α antagonist etanercept	▪ 50% PWT ▪ SF ▪ SG ▪ LUS ▪ Forced ambulatory guarding	*Spinal cord L4* GFAP (IF) *Femur tissue* Mac1, TNF-α (IF) Bone radiographs and histology	*After TCI* ↑ TNF-α (macrophages ++) ↑ GFAP (astrocyte activation) ↑ bone cancer-related pain behaviors *After blocking TNF-α* ↓ bone cancer-related pain behaviors *In TNFR1* + *2ko mice* ↓ mechanical allodynia ↓ astrocyte activation ↑ spontaneous pain (SG) ↑ tumor growth

Legend: *BCP* bone cancer pain, *β-EP* beta-endorphin, *β-tubulin* neuron marker, *BCP* cancer-induced bone pain, *CCP* conditioned place preference to pain relief by saphenous lidocaine (nerve block), *CCR2* chemokine receptor 2, *CD11b* microglia marker, *c-Fos* proto-oncogene (immediate-early gene) expressed in neurons upon neuronal activation, *CXCL1* chemokine (C-X-C motif) ligand 1, *CXCR2* C-X-C chemokine receptor type 2, *CXCL10* C-X-C motif chemokine 10, *CXCR3* C-X-C chemokine receptor type 3, *CXCL12* C-X-C motif chemokine 12, *CXCR4* C-X-C chemokine receptor type 4, *CXCL13* C-X-C motif chemokine 13, *CXCR5* C-X-C chemokine receptor type 5, *dlPAG* dorsolateral periaqueductal gray, *DRG* dorsal root ganglion, *ELISA* enzyme-linked immunosorbent assay, *Foxp3* transcription factor forkhead box P3 Treg cell marker, *GFAP* glial fibrillary acidic protein, a marker of astrocytes, *GM-CSF* granulocyte–macrophage colony-stimulating factor, *HMGB1* high mobility group box 1, *Iba-1* ionized calcium-binding adaptor molecule 1, a marker of microglia, *IF* immunofluorescence, *IFN-γ* interferon-gamma, *IL* interleukin, *IL-1RI* interleukin 1 receptor type I, *JNK* c-Jun N-terminal kinase, *ko* knockout, *LUS* limb use score, *LV* lentivirus, *Mac1* macrophage marker, *MCP-1* monocyte chemoattractant protein-1, *MWD* mechanical withdrawal duration, *NeuN* neuronal nuclei, neuronal marker, *NF-kB* nuclear factor kappa B, *NLRP3* leucine-rich repeat and pyrin domain containing protein 3, *NMDAR1,NR1 N*-methyl-d-aspartate receptor subunit 1, *PAG* periaqueductal gray, *PI3K* phosphoinositide 3-kinase, *PIC* pro-inflammatory cytokines, *PWT* paw withdrawal threshold, *PWL* paw withdrawal latency, *RAGE* receptor for advanced glycation end products, *RT-PCR* reverse transcription polymerase chain reaction, *SD* Sprague–Dawley, *SDH* spinal dorsal horn, *SDF-1* stromal cell-derived factor 1, *SF* spontaneous flinching, *SFI* sciatic functional index, *SG* spontaneous guarding, *SOCS3* suppressor of cytokine signaling 3, *ST2* suppressor of tumorigenicity 2, receptor for interleukin 33, also known as IL1RL1(interleukin 1 receptor-like 1), *TCI* tumor cell implantation, *TLR4* Toll-like receptor 4, *TNF-α* tumor necrosis factor-alpha, *TNFR1* tumor necrosis factor receptor 1, *TNFR2* tumor necrosis factor receptor 2, *TRPA1* transient receptor potential ankyrin 1, *vlPAG* ventrolateral periaqueductal gray, *WB* western blot, *WBD* hind limb weight-bearing difference, *WT* wild type, ++ location where mainly expressed

The molecular targets investigated encompassed a range of inflammatory cytokines, such as tumor necrosis factor-alpha (TNF-α) [20–22, 44], granulocyte–macrophage colony-stimulating factor (GM-CSF), and a variety of interleukins including IL-1β [21, 40], IL-6 [21, 22, 41], IL-17 [42], IL-18 [23], IL-33 [43], and IL-24 [24]. A variety of chemokines and their receptors were also analyzed: CCL2/CCR2 [26, 27], CXCL1/CXCR2 [28, 29], CXCL10/CXCR3 [30], CXCL12/CXCR4 [31–33], CXCL13/CXCR5 [34, 35].

Pain behavior assessments involved measuring mechanical allodynia through paw withdrawal threshold (PWT) in response to the stimulation with von Frey filaments in most studies (*n* = 20) [20–30, 32, 34, 35, 37, 38, 40–43]. Five studies [31, 33, 36, 39, 44] utilized the 50% PWT (50% paw withdrawal threshold) meaning the mechanical force required to elicit a paw withdrawal response in 50% of animals.

Thermal hyperalgesia was assessed according to paw withdrawal latency (PWL) in ten studies [21–23, 25, 29, 31–33, 36, 43]. Other pain behavior assessment methods were also used, such as spontaneous pain (through guarding and spontaneous flinching, *n* = 4) [33, 42–44] or limb use score (*n* = 4) [23, 33, 43, 44] to evaluate movement-evoked pain.

Protein expression and localization were assessed in the dorsal root ganglia (DRG) (*n* = 3) [22, 32, 36], spinal cord segments (*n* = 18) [20, 23, 26, 27, 29–35, 37–40, 42–44], and periaqueductal gray (PAG) (*n* = 2) [21, 28], mostly through western blotting and immunofluorescence.

### Summary of key findings

All studies that evaluated PWT and PWL as pain behavior assessments demonstrated a decrease in these values in bone cancer models compared to control animals. Additionally, all other pain behavior indicators were increased after tumor cell implantation across all included studies.

Studies evaluating the expression of classic pro-inflammatory cytokines (TNF-α, IL-1β, IL-6, IL-17, IL-18, IL-33) in the spinal cord, PAG, and DRG revealed upregulation following inoculation of cancer cells to establish the BCP models [20–23, 27, 37–40, 42–44]. Interventions that inhibited these cytokines attenuated mechanical [20–23, 40–44] and thermal [21, 22, 43] allodynia and reduced other pain behaviors [23, 41–44]. Four studies targeted TNF or its receptor [20–22, 44] resulting in reduced pain behaviors. The inhibition of IL-1β signaling was the intervention in two studies [21, 40] leading to attenuated hypersensitive responses. Three studies focused their intervention on blocking IL-6 [21, 22, 41] resulting in pain reduction. Inhibiting IL-17 [42], IL-18 [23], and IL-33 [43] also suppressed pain behaviors. One study evaluated the possible role of IL-24, a member of the IL-10 family, in inhibiting cancer pain. It was demonstrated that IL-24 mediated by adenovirus could significantly attenuate BCP and increase β-endorphin levels while decreasing IL-6 concentration in the plasma of animals [24]. It was demonstrated that targeting GM-CSF significantly alleviated both mechanical and thermal hyperalgesia [25].

Chemokines and their role were also evaluated in several studies of this review. In BCP models, the expression of all chemokines assessed CCL2/CCR2 [26, 27], CXCL1/CXCR2 [28, 29], CXCL10/CXCR3 [30], CXCL12/CXCR4 [31–33], and CXCL13/CXCR5 [34, 35] was increased in the spinal cord after BCP induction. Targeting these molecules attenuated pain hypersensitivity consistently throughout every study.

One study’s findings include the significant downregulation of SOCS3 in DRG following tumor cell injection, and subsequent overexpression attenuated pain hypersensitivity probably via reversing TLR4 upregulation [36].

Another study demonstrated that after TCI, the expression of NLRP3 inflammasome was upregulated in the spinal cord. Treatment of the NLRP3 inhibitor MCC950 markedly alleviated BCP-related mechanical allodynia suppressing the activation of NLRP3 inflammasome and spinal inflammatory cytokines in BCP rats [37].

Two studies focused on HMGB1, finding that spinal HMGB1 upregulation contributes to bone cancer pain and that treatment with anti-HMGB1 significantly reversed bone cancer-induced mechanical allodynia [38, 39].

Several studies implicated the involvement of glial cells, such as microglia and astrocytes, in the modulation of BCP [20, 23, 28, 30, 32, 38, 42, 43].

### Risk of bias assessment

**Fig. 3:**
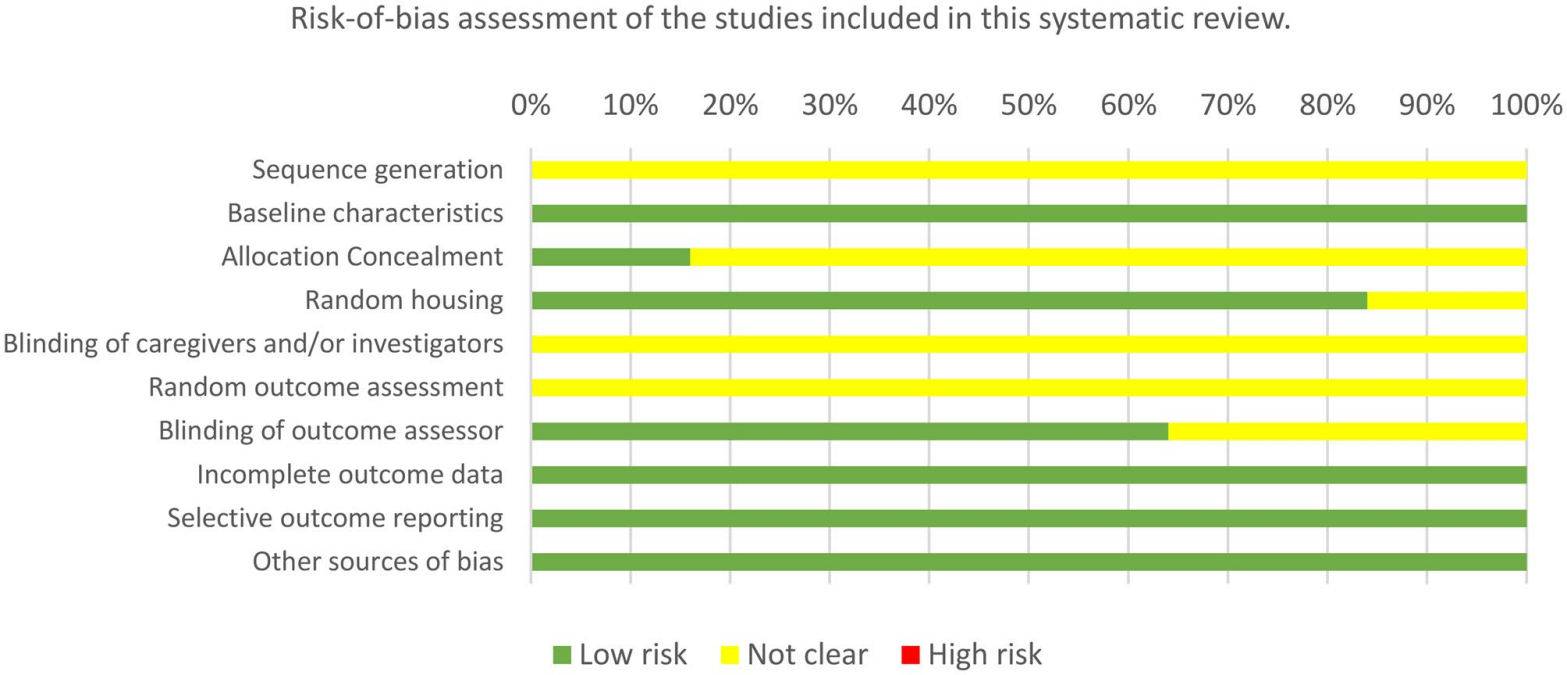
Risk of bias assessment according to SYRCLE tool per domain

Regarding the assessment of the risk of bias (Table 2; Fig. 3), using the SYRCLE tool, no study clearly detailed the methods used to generate a random allocation of animals. In terms of baseline characteristics, all studies exhibited similar starting conditions. Four studies were clear about allocation concealment. Twenty-one studies indicated randomized housing. None of the studies reported blinded allocation or randomization of outcome evaluation. Sixteen studies indicated blinding of the outcome assessor. All these studies have comprehensive outcome-based data and published intended results. Concerning other sources of bias, all studies showed no conflict of interest between authors.

**Table 2:**
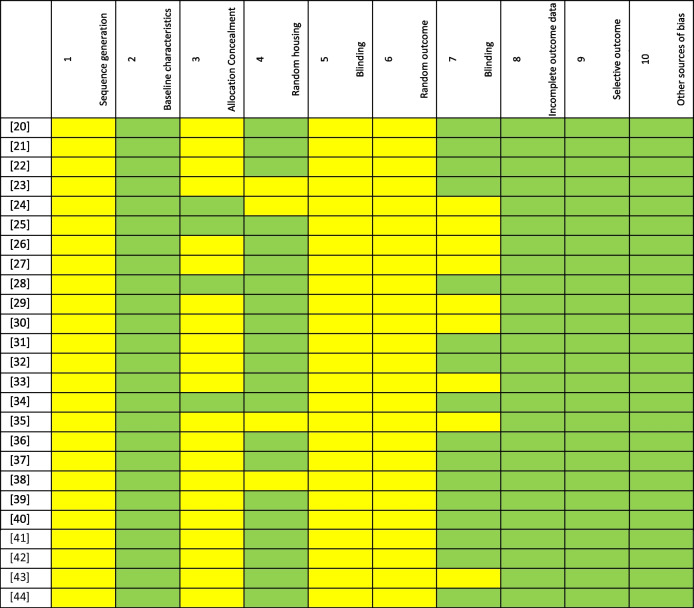
The risk of bias in the individual animal studies included

Legend: Green color: low risk, Yellow color: not clear

Since many items were not explained in detail in the included studies, this leads to great difficulties and deviations in the interpretation of research bias. Nonetheless, the overall risk of bias was considered low to moderate.

## Discussion

The main results indicate that targeting various inflammatory pathways shows promising results in alleviating BCP, mainly through the inhibition of pro-inflammatory cytokines and chemokines. Furthermore, IL-24 and SOCS3 demonstrated pain relief effects by modulating inflammatory mediators, and glial cells were identified as significant contributors to the development of BCP.

Among pro-inflammatory cytokines, TNF-α is recognized as a potent cytokine, rapidly produced, mainly by macrophages, leading to an increase in its levels in response to inflammatory stimuli or injury [45]. TNF-α interacts with target cells through high-affinity membrane receptors, namely TNF receptor type 1 (TNFR1) and type 2 (TNFR2) [46]. TNF-α triggers cytokine cascades, particularly IL-1β, IL-6, and IL-8 [47], driving both inflammatory and neuropathic hyperalgesia [48–57]. Its correlation with pain is noted across different conditions [58–61], and it also influences cancer-related chronic inflammation and progression [62, 63]. XPro1595, a TNF-α inhibitor, dose-dependently reduced mechanical allodynia in bone cancer rats, accompanied by decreased astrocyte and microglia activation and diminished pro-inflammatory cytokine production [20]. Injection of PTX (TNF-α synthesis inhibitor, pentoxifylline) and TRPA1 modulation attenuated mechanical and thermal hypersensitivity induced by bone cancer [22]. TNF-α (and IL-6) has a role in modifying the TRPA1 signal pathway [22]. TNF-α inhibition with etanercept attenuates pain response, achieved through downregulation of PI3K-mTOR expression in the dlPAG [21]. The combined absence of TNFR1 and TNFR2 attenuates cancer-related pain and concomitant spinal astrogliosis. However, TNF-α has the capability to promote tumor growth [44]. Nevertheless, exploring TNF-α’s mechanisms could unveil new targets, and combination therapies may provide comprehensive pain control with fewer side effects. Overall, TNF-α-targeted strategies hold potential for improving cancer pain management in the future personalized medicine approach.

In cancer pain, key ILs implicated include IL-1, IL-6, IL-10, IL-17, IL-18, and IL-33. These ILs contribute to the inflammatory responses and pain modulation associated with malignancy. IL-1β is acknowledged as an inflammatory mediator, mainly released by monocytes and macrophages, along with nonimmune cells like endothelial cells and fibroblasts [64]. The upregulation of IL-1β has been reported in the spinal cord during both inflammatory [65, 66] and neuropathic pain [67, 68], playing a role in facilitating the transmission and processing of noxious inputs at the spinal level [66, 69–71]. In BCP, IL-1β is consistently upregulated after tumor cell inoculation [20, 21, 34, 38–40, 43]. Spinal IL-1β increases NR1 phosphorylation to facilitate pain, and the administration of anakinra, an IL-1RI receptor antagonist, significantly reduces mechanical hyperalgesia and NR1 phosphorylation [40]. Inhibition of the IL-1β receptor relieved mechanical and thermal hyperalgesia in BCP, accompanied by downregulation of PI3K-mTOR [21].

IL-6 is recognized for its involvement in promoting pain by sensitizing nociceptors and amplifying signaling at the site of injury or disease. Its causal role extends to chronic inflammatory and immune diseases [72, 73], playing a crucial role in the pathogenesis of neuropathic pain, inflammatory pain, and BCP [74]. IL-6 also exhibits significant pro-tumorigenic activity, impacting bone metabolism, tumor cell proliferation and survival, angiogenesis, and inflammation [75]. During tumor growth, IL-6 was elevated in the plasma. Additionally, acute IL-6 signaling blockade with TB-2–081, an IL-6 signaling antagonist, demonstrated partial relief only in mechanical allodynia. On the other hand, continuous IL-6 blockade from tumor onset also effectively decreased ongoing pain and tumor-induced bone remodeling [41]. Administration of SC144, a complexed IL-6R-gp130 inhibitor, alleviated mechanical and thermal hypersensitivity in BCP rats [21, 22], reversing the upregulation of TRPA1, p-p38-MAPK, p-JNK [22], and PI3K-mTOR [21] induced by cancer. Crafting tailored treatments centered on IL suppression, such as utilizing tocilizumab and anakinra, holds the potential for more precise and efficient pain management. Tailoring treatments based on individual IL levels and responses could optimize pain relief for patients.

Numerous cytokines are recognized for their ability to induce chemotaxis; a specific subgroup of structurally related cytokines is known as chemokines. While their primary role involves recruiting white blood cells to the inflammation site, chemokines also play additional roles in angiogenesis and immune response, also contributing to the regulation of fever [76]. Chemokine receptors are extensively distributed in white blood cells, neurons, and glial cells [77]. It appears that chemokines play a role in enhancing pain sensitivity and spontaneous pain, either through direct action or by modulating the activity of nociceptors [78–80]. An important chemokine, MCP-1 (also known as CCL2), induced mechanical allodynia in naïve rats, and CCR2 antagonist RS-504393 reduced pain sensitivity [26]. PI3K/Akt [26] and NF-kB signaling pathways are involved in the regulation of the MCP-1/CCR2 axis in the spinal cord of BCP rats, contributing to the maintenance of pain by influencing the inflammatory process [27]. PAG administration of exogenous CXCL1 induced mechanical allodynia, while neutralizing antibodies against CXCR2 and CXCL1 attenuated BCP. NF-κB is implicated in the production of CXCL1 in astrocytes, and its inhibition results in decreased mechanical allodynia [28]. JNK activation is an upstream step in the production of CXCL1 in BCP [29]. CXCL10/CXCR3 signaling is also a contributor to the development and maintenance of BCP, most likely through microglial activation [30]. CXCL12/CXCR4 pathway leads to BCP through the activation of astrocytes and microglia and sensitizing neurons [31–33]. Spinal RhoA/ROCK2 pathway [31] and CaMKII/CREB pathway [33] have been identified as key downstream targets in CXCR4-mediated hyperalgesia and neuronal sensitization. The role of chemokine CXCL13 is particularly interesting; besides contributing to the development of BCP in rats, CXCL13 acts as a negative regulator in morphine analgesia [34, 35], possibly via p38, ERK, and AKT pathway [35]. Blocking CXCL13 signaling may therefore be a target to improve morphine analgesia in patients with cancer pain.

In the central nervous system, neurons and glial cells interact intricately to process and modulate pain, playing a crucial role in both acute and chronic pain states, particularly in BCP [20, 32, 42, 66, 68, 69, 81, 82]. Following tumor cell implantation, microglia and astrocytes become activated, manifesting as microgliosis and astrogliosis. Activated glial cells release numerous pro-inflammatory mediators, upregulate cell surface receptors, and activate intracellular signaling pathways. These processes enhance pain sensitivity and persistence. Activation of spinal cord microglia via the neuronal complement pathway (involving C1, C2, and C3) through the complement 3 receptor (C3R) enhances pain sensitivity [83]. Astrocytes contribute via the GABAergic pathway, leading to disinhibition and heightened pain sensitivity [84]. Additionally, the CXCR4-RhoA/ROCK2 signaling pathway in spinal neurons, activated by increased CXCR4 expression, promotes glial activation and pain hypersensitivity [31]. Microglial activation involves the release of pro-inflammatory cytokines like IL-18, which activate NMDA receptors on neurons, enhancing pain signals [23]. The P2X7 receptor on microglia triggers the NLRP3 inflammasome, releasing IL-1β and exacerbating pain through inflammation [85]. These complex neuron-glial interactions induce long-term neuronal changes, enhancing excitability and promoting sensitization, underscoring the potential for targeting glial pathways in developing effective BCP treatments, necessitating further research to optimize therapeutic strategies.

When it comes to studies in humans, there is still a significant journey ahead to better understand the link between inflammation and BCP. It was demonstrated that women with advanced breast cancer frequently experience pain and have high systemic levels of TNF-α and IL-1β [86]. A positive correlation between increased IL-6 levels and pain intensity in cancer patients undergoing chemotherapy has been found in clinical practice [87]. Recently, an exploratory analysis of serum cytokines in 57 metastatic breast cancer patients identified nine cytokines (GM-CSF, IFNγ, IL-1β, IL-2, IL-4, IL-5, IL-12p70, IL-17A, and IL-23) that could best predict pain severity [88].

Therapeutic translation is always deeply challenging, particularly in the case of the pathophysiologically diverse condition that is BCP. Despite encouraging outcomes in animal models, targeting pro-inflammatory cytokines and chemokines has not yet been properly studied in clinical trials of BCP. Nevertheless, clinical studies in other chronic pain conditions have supported the analgesic potential of suppressing pro-inflammatory cytokines, such as IL-6 and TNF-α [89–93]. Two cases have been reported where targeted administration of etanercept in an anatomical site proximal to bone metastasis in patients experiencing refractory pain resulted in prompt, significant, and prolonged relief of their complaints. This relief is most likely linked to the role of TNF-α in inhibiting osteoclast-mediated bone reabsorption [94].

The evidence under review suggests that pharmacological interventions targeting inflammatory cytokines hold promise for managing pain in advanced cancer patients struggling with BCP. However, preserving normal cytokine function is crucial, through a delicate balance between mitigating excessive inflammation and maintaining vital immune response and homeostasis [95]. The challenge in administering cytokine or chemokine inhibitors lies in their pleiotropic nature; any cytokine-targeted therapy may act as a double-edged sword, with both beneficial and detrimental effects on human health [96–98].

Something worth considering in clinical BCP research is stratifying human patient populations based on excessive/dysregulated inflammatory responses. Identifying inflammatory biomarkers holds the potential to discern patients who might benefit from targeted cytokine therapies, a pivotal step toward precision medicine [99, 100]. Apart from directly measuring cytokine levels, it is important to note that C-reactive protein (CRP) also acts as a biomarker for systemic inflammation and reflects IL-6 levels since its production depends on IL-6 [99, 101].

To bridge the gap between animal and clinical pain research in BCP, preclinical studies must be more rigorous, and animal models should closely replicate human pathology. Using markers of bone remodeling and imaging techniques from human studies can enhance model validity. Due to the varied manifestations of BCP across different cancers and metastasis sites, multiple animal models are necessary to explore variations in pain etiology and drug responsiveness. Effective BCP management involves polypharmacy or agents with polypharmacology. Align animal models with human BCP is essential to identify clinically efficacious agents and compatibility with adjunct therapies. Chemotherapy’s modulation and its potential impact on immune-targeting BCP interventions highlight the need for evaluating therapeutic compatibility in preclinical studies. Given the interrelation between pain, bone wasting, and tumor burden, pharmacological interventions must address the multiple aspects of BCP [15, 16, 18].

Previous reviews have explored the association between cytokines and chemokines with pain [102–105], including specific molecules such as CXCL3 [106] or IL-17 [107]. Furthermore, cytokines and chemokines can be potential targets for glial cell modulation [82] and morphine tolerance management [108] in chronic pain states. However, the present review specifically targets BCP, providing a comprehensive examination of the unique pathophysiological mechanisms and potential therapeutic targets associated with this condition. Additionally, it also underscores the significant role of glial cells in releasing inflammatory cytokines. Moreover, the present review emphasizes the promise and challenges of translating these findings into effective therapeutic strategies, bridging the gap between preclinical findings and clinical advancements, ultimately aiming to enhance patient quality of life.

The limitations of this research included only animal studies, as the extent to which findings in animal models mirror the human experience of BCP remains a critical question. Differences in disease pathophysiology, host responses, and genetic diversity between rodents and humans limit the applicability of animal models to human BCP. Animal studies often focus on reflexive behaviors, which may not capture the chronic and complex nature of human BCP. Furthermore, variations in treatment responses between species necessitate careful validation of preclinical findings in human clinical trials to ensure translational relevance [18]. Additionally, the research methods of the included manuscripts were conducted in rodent models with samples collected from the CNS, which makes it difficult to extrapolate findings to humans. It should also be considered that the studies were conducted in rats of only one sex, either male or female, and none of them investigated both sexes simultaneously. A meta-analysis was not possible to perform due to the high variability of the experimental protocols included in this study. These protocols often lack details about the methodology used, a problem previously identified in animal studies by the authors of the SYRCLE tool themselves. Nevertheless, there was no high risk of bias in the included studies.

Future research should prioritize establishing standardized methodologies, outcome measures, and reporting practices. Bridging the translational gap between preclinical findings and clinical applications remains crucial, necessitating robust investigations into the relevance of identified molecular targets in human BCP. Longitudinal studies exploring neuroinflammatory changes at different stages of cancer progression are vital for a comprehensive understanding of BCP. Moreover, considering the complex interplay of multiple molecular targets, research on combinatorial therapeutic approaches tailored to individual molecular profiles holds promise for more effective BCP management in the era of personalized medicine.

## Conclusions

Based on the comprehensive assessment provided in this systematic review, targeting inflammatory cytokines holds significant promise in managing BCP, as demonstrated in animal models. Glial cells, due to their involvement in the release of inflammatory cytokines, emerged as significant contributors to BCP. Targeting specific cytokine pathways, such as TNF-α, IL-6, and IL-1β, highlights the potential for new therapeutic options, ultimately leading to an enhanced quality of life for individuals grappling with the debilitating symptoms of BCP. This work underscores the importance of continued research in this area to translate these findings into effective clinical treatments. As we move forward, these findings imply a paradigm shift toward personalized, precision medicine approaches, fostering optimism in the pursuit of more effective therapeutic strategies.

## Data Availability

All data generated or analyzed during this study are included in this article.
